# Results of Four-Year Rectal Vancomycin-Resistant Enterococci Surveillance in a Pediatric Hematology-Oncology Ward: From Colonization to Infection

**DOI:** 10.4274/tjh.2015.0368

**Published:** 2016-08-19

**Authors:** Hacer Aktürk, Murat Sütçü, Ayper Somer, Serap Karaman, Manolya Acar, Ayşegül Ünüvar, Sema Anak, Zeynep Karakaş, Aslı Özdemir, Kutay Sarsar, Derya Aydın, Nuran Salman

**Affiliations:** 1 İstanbul University İstanbul Faculty of Medicine, Department of Pediatric Infectious Diseases, İstanbul, Turkey; 2 İstanbul University İstanbul Faculty of Medicine, Department of Pediatric Hematology and Oncology, İstanbul, Turkey; 3 İstanbul University İstanbul Faculty of Medicine, Infection Control Committee, İstanbul, Turkey; 4 İstanbul University İstanbul Faculty of Medicine, Department of Clinical Microbiology, İstanbul, Turkey

**Keywords:** Colonization, Infection, Pediatric malignancy, Vancomycin-resistant enterococci

## Abstract

**Objective:**

To investigate the clinical impact of vancomycin-resistant enterococci (VRE) colonization in patients with hematologic malignancies and associated risk factors.

**Materials and Methods:**

Patients colonized and infected with VRE were identified from an institutional surveillance database between January 2010 and December 2013. A retrospective case-control study was performed to identify the risk factors associated with development of VRE infection in VRE-colonized patients.

**Results:**

Fecal VRE colonization was documented in 72 of 229 children (31.4%). Seven VRE-colonized patients developed subsequent systemic VRE infection (9.7%). Types of VRE infections included bacteremia (n=5), urinary tract infection (n=1), and meningitis (n=1). Enterococcus faecium was isolated in all VRE infections. Multivariate analysis revealed severe neutropenia and previous bacteremia with another pathogen as independent risk factors for VRE infection development in colonized patients [odds ratio (OR): 35.4, confidence interval (CI): 1.7-72.3, p=0.02 and OR: 20.6, CI: 1.3-48.6, p=0.03, respectively]. No deaths attributable to VRE occurred.

**Conclusion:**

VRE colonization has important consequences in pediatric cancer patients.

## Abstract (tr)

**Amaç:**

Hematolojik malignitesi olan pediatrik hastalarda vankomisine dirençli enterokok (VDE) kolonizasyonunun klinik öneminin araştırılması ve eşlik eden risk faktörlerinin değerlendirilmesi amaçlanmıştır.

**Gereç ve Yöntemler:**

Ocak 2010-Aralık 2013 tarihleri arasında yapılan VDE sürveyans kayıtlarından faydalanılarak VRE ile kolonize ve/veya enfekte olmuş hastalar belirlenmiştir. VDE ile kolonize hastalarda VRE enfeksiyonu gelişimi ile ilişkili risk faktörlerini belirlemek amacıyla retrospektif olgu-kontrol çalışması yapılmıştır.

**Bulgular:**

Çalışma süresince yatırılan 229 hastanın 72’sinde (%31,4) rektal VRE kolonizasyonu saptanmıştır. VRE kolonize hastaların yedisinde (%9,7) kolonizasyon sonrası sistemik VRE enfeksiyonu gelişmiştir. Beş hastada bakteriyemi (n=5), bir hastada idrar yolu enfeksiyonu (n=1) ve bir hastada menenjit (n=1) gözlenmiştir. Tüm enfeksiyonlarda ilgili klinik örneklerde Enterococcus faecium üretilmiştir. Multivaryant analiz sonucunda, ciddi nötropeni ve başka bir patojene bağlı bakteriyemi öyküsü VDE kolonize hastalarda VDE enfeksiyonu gelişimi için risk faktörleri olarak bulunmuştur [odds ratio (OR): 35,4; güven aralığı (GA): 1,7-72,3; p=0,02 ve OR: 20,6; GA: 1,3-48,6; p=0,03; sırasıyla]. VDE enfeksiyonuna bağlı mortalite saptanmamıştır.

**Sonuç:**

Pediatrik kanser hastalarında VDE kolonizasyonunun önemli klinik sonuçları olduğu gözlenmiştir.

## INTRODUCTION

Children with cancer are at high risk of developing systemic infections by the microorganisms that colonize their own intestinal system [1,2]. Vancomycin-resistant enterococci (VRE) are health care-associated opportunistic pathogens. Limited data exist on the incidence of subsequent VRE infection development among VRE-colonized pediatric cancer patients and associated risk factors, which were investigated in this study.

## MATERIALS AND METHODS

All patients admitted to the pediatric hematology/oncology ward were sampled within 48-72 h after admission and weekly thereafter as part of institutional rectal VRE surveillance. An infection control nurse assigned by the Hospital Infection Control Committee (HICC) prospectively tracked the results of rectal surveillance and all health care-associated infections occurring in the hematology/oncology ward. VRE-colonized and VRE-infected patients were identified from the HICC surveillance database retrospectively. Detailed clinical and laboratory features of these patients were collected from their medical records. The overall rate of VRE colonization and the subsequent infection occurrence throughout the study period were determined. To identify the risk factors associated with VRE infection occurrence in a colonized patient, a retrospective case-control study was performed. Patients were defined as VRE-colonized (VRE-C) when the culture of the rectal swab yielded VRE in the absence of any clinical specimens positive for VRE [3]. Systemic VRE infection (VRE-I) was defined as isolation of VRE from a clinical specimen together with signs and symptoms of infection. Statistical analysis was performed with SPSS 21.0 for Windows. Parameters were compared between groups with the chi-square test, Fisher exact test, or Mann-Whitney U test. Variables with a p-value of ≤0.1 in univariate analysis were fitted to perform logistic regression analysis to identify independent risk factors associated with VRE infection occurrence.

## RESULTS

A total of 229 children were admitted to the hematology/oncology ward. Fecal VRE-C was documented in 72 of these patients (31.4%). Excluding eight patients who were transferred from the pediatric intensive care unit, 89% of the patients were colonized during their stay in the hematology/oncology ward. Species determination could be performed in 32 VRE-colonized patients: Enterococcus faecium was isolated in 28 patients, Enterococcus gallinarum in 2 patients, and nontypeable Enterococcus in 2 patients.

VRE-I was detected in 7 patients, all of whom were previously colonized with VRE. The overall rate of VRE-I developing in patients with VRE-C was 9.7%. VRE bacteremia was detected in five patients (6.9%). Other VRE infections were urinary tract infection in one patient and meningitis in one patient. Enterococcus faecium was isolated in all patients with VRE-I. The mean duration of time from identification of VRE colonization to development of a VRE infection was 32.4±8.6 days (median: 25 days, range: 10-73 days).

Univariate analysis of demographic and clinical variables associated with development of VRE-I among patients with VRE-C is presented in Table 1. Duration of neutropenia was significantly longer in patients with VRE-I than in patients with VRE-C (12.8±1.4 days vs. 38.5±7.3 days; p=0.016). Similarly, total parenteral nutritional support was found to be received for a longer time by patients with VRE-I (9.37±0.8 days vs. 15.33±5.5 days; p=0.04). Antimicrobials used among patients are shown in Table 2. Four out of seven patients were receiving a glycopeptide antibiotic when a systemic VRE infection was diagnosed. Multivariate analysis revealed severe neutropenia and history of previous bacteremia with another pathogen as independent risk factors for development of VRE infection in a VRE-colonized patient with cancer (Table 3).

All VRE infections were treated with linezolid. No VRE-attributable deaths occurred during systemic VRE infections. However, crude mortality was higher in patients who suffered from VRE infection than those who did not (2/7 (28.6%) vs. 4/65 (6.2%), respectively; p=0.04).

## DISCUSSION

Overall, 31.4% of patients admitted to our hematology/oncology ward were colonized with VRE. Colonization rates in other studies ranged from 4.7% to 38% [4,5,6,7]. Systemic VRE infections develop mostly in VRE-colonized patients [8,9], although some contrary cases may exist rarely [5,10]. In this study, about 1 in 10 VRE-colonized patients developed subsequent systemic VRE infection (9.7%). Studies evaluating cancer patients reported a range of 13% to 61% rate of progression of VRE colonization to VRE bacteremia [6,10,11,12,13].

Results of univariate analysis revealed some risk factors associated with VRE-I development. One of them was severe neutropenia (<100/mm^3^) and longer duration of neutropenia, which was consistent with the literature [4,7,12,13]. The presence and a longer duration of total parenteral nutritional support were more likely to be present in VRE-I patients. It increases the risk of infection due to the use of an invasive central line, tendency for a longer hospitalization, and disadvantageous effects on gastrointestinal flora [14,15]. Its association with VRE infection in our univariate analysis may merely indicate illness severity of the patients, which is the main predisposing factor for VRE bacteremia [10,16]. In case-control studies, colonization and infection with VRE have been associated with exposure to several antibiotics, especially to glycopeptides [8,17]. In the current study, VRE-I patients had longer duration of glycopeptide treatment and more frequently received glycopeptides after detection of VRE colonization, similar to literature findings [7,10,13]. After multivariate analysis, severe neutropenia and history of previous bacteremia with another pathogen remained as independent risk factors for development of VRE infection in a colonized patient. Previous bacteremia episodes were not evaluated in similar reports. It may be proposed that a previous blood stream infection history indicates the patient’s severity of illness, which in turn increases the risk of a VRE infection. Moreover, a bacteremia episode itself might have caused clinical deterioration of the patients, predisposing them to the progression of VRE colonization to a systemic infection.

It was reported that active screening leads to reduced VRE colonization, infection, and reduced costs [18]. In a comparative study, the rate of VRE infection was found to be lower in a hospital with active screening [19]. It increases awareness of the staff and leads to improvement in compliance with control measures. Thus, by detecting all carriers, onward transmission of colonization may be prevented and resultant VRE infections may be reduced. Our study revealed a remarkable incidence of VRE colonization and subsequent progression to infection, indicating the ongoing clinical importance of VRE in this high-risk population.

## CONCLUSION

This study provides the four-year incidence of VRE colonization and its clinical impact on pediatric malignancy patients. Furthermore, it evaluates risk factors for progression from colonization to infection, which may help physicians to identify VRE-colonized patients at high risk of developing systemic infection.

## Ethics

Ethics Committee Approval: The study protocol was approved by Ethics Committee of İstanbul University, İstanbul Medical Faculty; Informed Consent: Not required.

## Figures and Tables

**Table 1:**
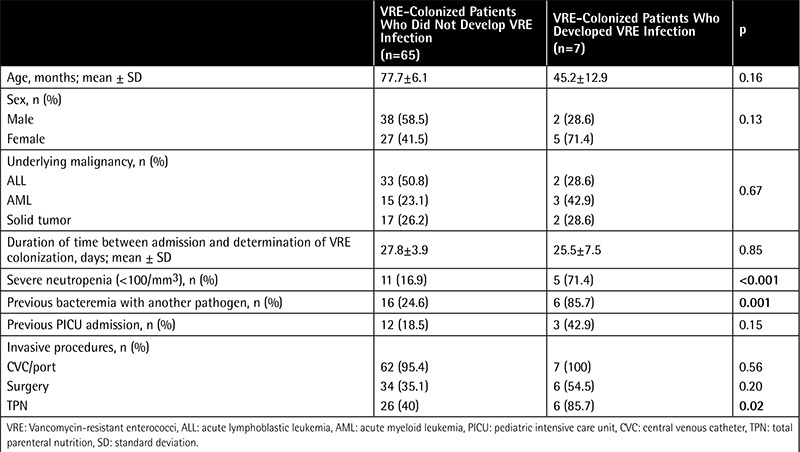
Demographic and clinical characteristics of vancomycin-resistant enterococci-colonized patients who developed systemic vancomycin-resistant enterococci infection and those who did not.

**Table 2:**
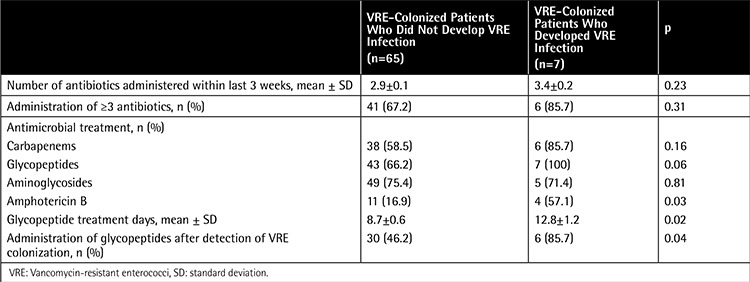
Antimicrobial use in vancomycin-resistant enterococci-colonized patients who developed systemic vancomycin-resistant enterococci infection and those who did not.

**Table 3:**
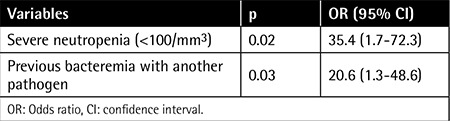
Multivariate analysis of risk factors for development of vancomycin-resistant enterococci infection in patients colonized with vancomycin-resistant enterococci.
